# Supranutritional Selenium-Yeast Supplementation of Beef Cows during the Last Trimester of Pregnancy Results in Higher Whole-Blood Selenium Concentrations in Their Calves at Weaning, but Not Enough to Improve Nasal Microbial Diversity

**DOI:** 10.3390/ani12111360

**Published:** 2022-05-26

**Authors:** Jean A. Hall, Anitha Isaiah, Ened R.L. McNett, Joseph J. Klopfenstein, T. Zane Davis, Jan S. Suchodolski, Gerd Bobe

**Affiliations:** 1 Department of Biomedical Sciences, Carlson College of Veterinary Medicine, Oregon State University, Corvallis, OR 97331, USA; mcnette@oregonstate.edu; 2 Gastrointestinal Laboratory, Department of Small Animal Clinical Sciences, College of Veterinary Medicine, Texas A&M University, College Station, TX 77843, USA; anitha.isaiah@gmail.com (A.I.); jsuchodolski@cvm.tamu.edu (J.S.S.); 3 Department of Clinical Sciences, Carlson College of Veterinary Medicine, Oregon State University, Corvallis, OR 97331, USA; joe.klopfenstein@oregonstate.edu; 4 USDA-ARS-Poisonous Plant Research Lab, Logan, UT 84341, USA; zane.davis@ars.usda.gov; 5 Department of Animal and Rangeland Sciences, College of Agricultural Sciences, Oregon State University, Corvallis, OR 97331, USA; gerd.bobe@oregonstate.edu; 6 Linus Pauling Institute, Oregon State University, Corvallis, OR 97331, USA

**Keywords:** cattle, feedlot performance, fetal programming, nasal microbiome, selenium, selenium-yeast, weaned beef calves, whole-blood selenium

## Abstract

**Simple Summary:**

We previously showed that feeding selenium-enriched alfalfa hay to weaned beef calves diversified the nasal microbiome and improved health and growth in the feedlot, resulting in greater carcass weight and quality at slaughter. The objective of the current study was to see whether selenium supplementation of dams during various pregnancy trimesters similarly improves health and performance. Maternal supranutritional selenium supplementation during pregnancy increases selenium concentrations in calves, but is more effective the shorter the time period between maternal supplementation and assessment. Thus, calves should be fed selenium-enriched forages again at weaning in order to see a benefit in the nasal microbiome abundance and diversity, and consequently, in carcass measurements at slaughter.

**Abstract:**

We previously reported that feeding Se-biofortified alfalfa hay to weaned beef calves in a preconditioning program increases whole-blood Se (WB-Se) concentrations and nasal microbiome abundance and diversity during the preconditioning period, decreases morbidity and mortality during the feedlot period, and increases carcass weight and quality at slaughter. The objective of the current study was to see whether similar improvements can be achieved through Se supplementation of dams during various pregnancy trimesters. In a two-year experimental study, 80 Angus-cross cows received once-weekly Se-yeast boluses containing 105 mg of Se, during either the first (TR-1), second (TR-2), or third (TR-3) pregnancy trimester, or were not bolused (CTR). Whole-blood Se concentrations were higher from CTR, to TR-1, to TR-2, and to TR-3 in newborn calves (all *p* < 0.01). At weaning, only calves from TR-3 mothers had higher WB-Se concentrations compared with calves from CTR mothers (*p* = 0.02), and no significant differences in nasal microbiome abundance and diversity or nasal microbiota were observed. In the feedlot period, morbidity was low, and no differences were observed. At slaughter, no differences in carcass weight and quality were observed. In conclusion, Se supplementation of pregnant cows is effective for increasing WB-Se concentration of newborn calves, and the increase can be sustained until weaning for calves born to TR-3 dams. However, the increase in WB-Se concentrations is small and does not result in beneficial changes in the nasal microbiome. Thus, calves should be fed Se-biofortified forages again at weaning in a preconditioning program in order to diversify the nasal microbiome prior to entering the feedlot.

## 1. Introduction

Selenium (Se) in an essential trace mineral for cattle that plays an important role in growth and health. Therefore, Se-deficient soils represent unique challenges for cattle producers in terms of cattle management. Clinical Se deficiency, indicated by a whole-blood (WB) Se < 50 ng/mL, can lead to nutritional myodegeneration (white muscle disease) and death, especially during periods of high growth rates such as in young animals [1,2]. Subclinical Se deficiency or Se concentrations below the reference range, indicated by a WB-Se of 50–100 ng/mL, has been linked to poor growth and a higher incidence of subclinical diseases [3]. These consequences can be acutely problematic when additional stressors are present such as with weaning and transfer to a feedlot, which increase Se requirements [4]. The more producers can do prior to transport to support calves during this vulnerable transition period, the better the health and productivity of the calf, which ultimately translates to increased profits for the producer as a result of increased carcass weight and quality. Additionally, we are arguably living in a post-antibiotic era. The methods we have previously relied on to control diseases in high-stress environments, such as the feedlot, are becoming more regulated and less effective. Researchers, veterinarians, and producers need to work together to find nutrition and management innovations that support immune function and mitigate disease risk [5].

The Northwest region is among those regions with the lowest amounts of Se in soils and plants [1]. In most of the Pacific Northwest, the Se concentrations of forages are inadequate to prevent clinical Se deficiency in livestock [6,7]. To prevent Se-responsive subclinical diseases, Se is supplemented through a variety of methods including salt–mineral mixes that contain inorganic Se, Se-fortified forages (such as Se-fertilized alfalfa hay; the primary form of Se in such forages is organic selenomethionine), or Se boluses (containing inorganic selenite, or organic Se as selenized yeast); these are reviewed in Brummer et al. [8]. Supplementation dosages and periods vary. We are specifically interested in Se dosages at concentrations that are higher than currently recommended by the US-FDA for preventing clinical Se deficiency (i.e., supranutritional dosages), because we have observed, at those higher concentrations, improved production and fewer diseases in cattle and sheep, without adverse health outcomes. We have evaluated supranutritional Se supplementation in Se-replete cattle and sheep throughout production stages, as well as during specific high-demand stages (e.g., during the backgrounding period before transport to the feedlot, and during the last 2–3 months of gestation) and have observed production and immune function improvements with both strategies in animals at the highest supplementation levels [9,10,11,12,13,14,15,16,17].

A major cause of morbidity and mortality in U.S. feedlot cattle is bovine respiratory disease (BRD) complex [4,18]. We postulate that bolstering the immune response of feedlot cattle will improve economic returns by preventing BRD. In support, we have shown that feeding Se-biofortified alfalfa hay to weaned, Se-replete beef calves in a preconditioning program decreases morbidity and mortality during the feedlot period, leading to an increase in carcass weight and quality at slaughter [11,12]. In these preconditioned calves, positive outcomes were linked with an increase in nasal microbiome abundance and diversity during the backgrounding period [11,12].

The objective of the current study was to evaluate whether similar changes in calves can be achieved by administering Se boluses to their dams during different trimesters of pregnancy (first trimester: TR-1; second trimester: TR-2; third trimester: TR-3). We chose Se boluses rather than Se-biofortified alfalfa hay, as one can verify the amount of Se from selenized yeast ingested using boluses; however, this method is more labor-intensive than feeding Se-biofortified forages. Similar to our previous studies, we used Angus and Angus-cross cows that also had access to sodium selenate in a salt–mineral mix, at concentrations recommended by the US-FDA for preventing clinical Se deficiency. We hypothesized from our prior studies that Se requirements may differ among the three trimesters of pregnancy. Furthermore, we hypothesized that maternal Se supplementation may impact fetal programming (e.g., muscle growth, immune function, or nasal microbiome abundance and diversity). Selenium-responsive fetal programming may also impact outcomes long after the supplementation period has ended—e.g., changing calf growth and health prior to weaning as well as in the feedlot—and may affect carcass characteristics at slaughter. To our knowledge, this is the first study that examines the impact of maternal Se supplementation during different trimesters of pregnancy rather than focusing on the last weeks of pregnancy or throughout pregnancy.

## 2. Materials and Methods

### 2.1. Animal Ethics Statement and Study Design

**Figure 1:**
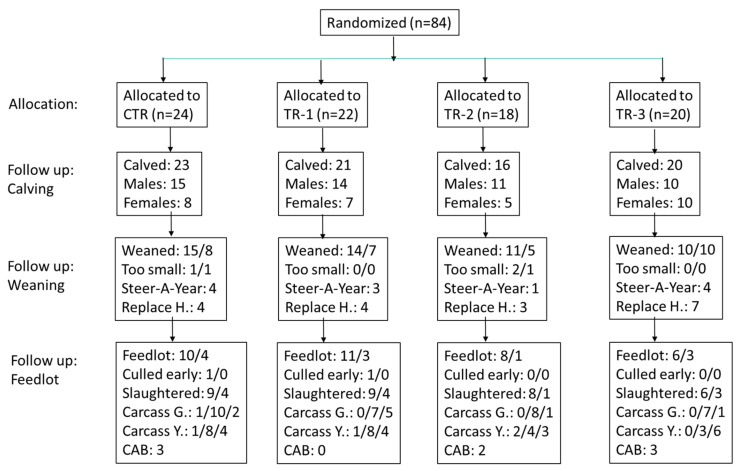
Flow chart of the maternal Se-supplementation study. For follow-up weaning and follow-up feedlot data, the number before the slash represents the number of males and the number after the slash represents the number of females. “Too small” refers to weaned calves that could not be sold to the feedlot because of their small size. “Steer-A-Year” refers to a beef production course that elected to keep some of the larger steers, and “Replace H.” refers to heifers that were kept by the Soap Creek Ranch for replacements. “Culled early” refers to calves in the feedlot that did not reach slaughter weight. “Carcass G.” refers to carcass grade, with the number before the slash reporting the number of calves graded “prime”; the second number is the number of calves graded “choice”; and the third number is the number of calves graded “select”. “Carcass Y.” refers to carcass yield, with the number before the slash reporting the number of calves with yield = 1; the second number is the number of calves with yield = 2; and the third number is the number of calves with yield = 3. “CAB” refers to “Certified Angus Beef^®^” with the number of calves attaining this quality certification so indicated.

The Oregon State University Animal Care and Use Committee reviewed and approved the experimental protocol (ACUP Number: 2019–0056). The study design was a prospective clinical trial of a 2-year duration (May 2019–June 2021), and included calves born to beef cows that were bolused once weekly with Se yeast during various trimesters of pregnancy (Figure 1). Cows were assigned to one of four groups at conception (control, and groups 1, 2, and 3, corresponding to trimester of Se treatment: CTR, TR-1, TR-2, and TR-3, respectively), using a randomized complete block design. The cows were Angus and Angus-cross cows that were housed at the Oregon State University Soap Creek Ranch in Corvallis, OR. Beef cows were between 3 and 13 years of age with a mean age of 6.1 years and a standard deviation of 2.24 years. All cows were in good body condition; 24 cows had small frame size, 48 cows had medium frame size, and 8 cows had large frame size. To achieve homogeneity in calf crop, we used sexed semen of one sire to artificially inseminate cows. Several bulls were used to inseminate cows that did not conceive the first time. Cows were identified by ear tags.

Forage Se concentrations in this region range from 0.06 to 0.11 mg/kg (DM basis) [19,20]. All cows and calves received the same routine farm management practices (e.g., feeding (pasture and hay), vaccinations, and deworming). All TR cows were Se supplemented during their corresponding pregnancy trimester in the form of three Se-yeast (Phibro Selenium Yeast 2000, Prince Agri Products, Inc., Quincy, IL, USA) boluses once per week for 13 weeks, equaling 105 mg Se/wk throughout their treatment trimester (TR-1, TR-2, or TR-3). The Se supplementation dosage was five times the upper limit for Se supplementation that the National Research Council (NRC) recommends, but well below the documented toxic threshold for ruminants (5 mg/kg is defined as the maximum tolerable level in ruminants) [21]. All cows had ad libitum access to a mineral supplement containing 120 mg/kg Se (US FDA regulations) from Na selenite.

After calving, all cows and calves were maintained on pasture and hay at the Soap Creek Ranch in Corvallis, OR, and had free-choice access to a mineral supplement containing 120 mg/kg Se from sodium selenite. Routine farm management practices, including vaccinations and deworming, were the same for all treatment groups. Health status was monitored during the pre-weaning period (e.g., days off feed, fever, respiratory disease, diarrhea, abscess, pink eye, etc.). The calves were weaned in October 2020 between 190 and 245 days of age, with a mean ± STDev of 224 ± 14 days. The best steers were kept for a beef production course (“Steer-a-Year”) and the best females were kept as replacement heifers. The remaining calves were shipped six weeks later (November 2020) to a commercial feedlot (Simplot Feeders, Pasco, WA, USA) in Burbank, WA. The feedlot rejected the smallest calves, which had to be sold privately. Calves were slaughtered in June 2021 at a commercial facility (Tyson Fresh Meats, Pasco, WA, USA) between 439 and 495 days of age, with a mean ± STDev of 474 ± 14 days, and carcass weight and characteristics were determined. Carcasses were weighed and graded. Eight carcasses were classified as “Certified Angus Beef^®^” (CAB). Besides consistent sizing, the CAB certification, indicating superior carcass quality, requires a maximum of 1-inch fat thickness, superior muscling, a lack of quality deficits, and a minimum of 5.8% evenly distributed intramuscular fat content of the LD muscle with medium-to-fine marbling texture.

### 2.2. Blood Se Collection and Analyses

Whole-blood samples were collected within 12 to 48 h of birth (day 0), at 14 days of age, at 60 days of age, and again at weaning, into evacuated 2-mL EDTA tubes (final EDTA concentration of 2 g/L; Becton, Dickinson and Company, Franklin Lakes, NJ, USA) and stored on ice until they were frozen at −20 °C. Frozen whole-blood samples were shipped on dry ice to Utah Veterinary Diagnostic Laboratory, a commercial laboratory in Logan, UT, to measure concentrations of WB-Se. In short, 750 µL of whole blood was mixed with 750 µL of trace-metal-grade nitric acid in a 10-mL digestion tube. Next, the blood was digested for 2 h at 90 °C in the cap-sealed tube. After cooling down, the contents were transferred to another trace-metal-free tube. Next, 1 mL of the digested blood was transferred into another trace-metal-free tube, which already contained 9.0 mL of ultrapure water, to form a 5% nitric acid matrix. After vortexing, the sample was analyzed on an inductively coupled argon plasma emission spectrometer (ICP-MS; ELAN 6000, Perkin Elmer, Shelton, CT, USA) using a 4-point standard curve. A pooled QC sample dissolved in a 5% nitric acid matrix was analyzed after every 5 samples. If the pooled QC sample fell within ±5% of the reference value, the WB-Se analysis was considered acceptable.

### 2.3. Nasal Microbiota Collection and Analyses

Based on the uniform genetic background of cows (black color, medium size, and middle aged (5 to 9 years)), ten calves were chosen from each group for assessment of nasal microbiota. The resulting calf group demographics from these cows were as follows: CTR group *n* = 10 (4 females, 6 males), TR-1 *n* = 9 (2 females, 7 males), TR-2 *n* = 11 (3 females, 8 males), TR-3 *n* = 10 (6 females, 4 males). Nasal swabs (polyester-tipped applicators; Puritan1, Guilford, ME, USA) were collected to assess the nasal microbiome at weaning (6.5 to 8 months of age). The nasal swabs, which were sterile and wrapped individually, were inserted approximately 10 cm into the ventral meatus of the nares, twirled to collect a mucosal swab, and then placed into individual sterile containers (10 mL, red top, BD Vacutainer collection tubes; Becton Dickinson, Franklin Lakes, NJ). Care was taken to avoid any contamination or human contact with the plastic stick. All swabs were then frozen at −80 °C within 4 h of collection in their collection tubes. Sterile swabs (negative controls) were processed in the same manner.

A MoBio Power soil DNA isolation kit (MoBio Laboratories, Carlsbad, CA, USA) was used per the manufacturer’s instructions, to extract microbial DNA from the nasal swabs. The V4 region of the 16S rRNA gene was amplified with primers 515F (50-GTGCCAGCMGCCGCGGTAA-30) and 806R (50-GGACTACVSGGGTATCTAAT-30) at the MR DNA Laboratory (Shallowater, TX, USA) and sequenced on an Illumina MiSeq instrument at the MR DNA Laboratory (Shallowater, TX, USA).

The QIIME 2 v 2018.6 (https://qiime2.org/, accessed on 25 May 2022) platform was used to process the sequencing data [22]. The raw reads were deposited in NCBI SRA under the accession number PRJNA821917. The q2-demux plugin in QIIME2 was used to de-multiplex the raw sequence data. The data were quality filtered by removing low-quality regions and chimeric sequences using the Divisive Amplicon Denoising Algorithm 2 (DADA2) with the q2-dada2 plugin [23] to create an amplicon sequence variant (ASV) table. MAFFT [24] with the q2-alignment plugin was used to conduct a masked alignment of sequence variants. A phylogeny tree was created with FastTree2 [25] using the q2-phylogeny plugin. Furthermore, taxonomy was assigned using the QIIME2 naive Bayes feature classifier [26] trained on the Greengenes 13_8 database [27]. The feature table was also filtered to remove sequences that were classified as mitochondria and chloroplasts.

### 2.4. Statistical Analyses

Analysis of WB-Se and body weight was performed using PROC MIXED in SAS version 9.2 [28]. Fixed effects in the model were maternal Se supplementation (CTR, TR-1, TR-2, and TR-3), sex, time, as well as the interaction between sex and time and between maternal Se supplementation and time. To account for repeated measurements within calves, we modeled within calf-variation using an unrestricted variance–covariance structure. For body weight data, we added cow frame (small, medium, large) and its interaction with time into the model. We checked posteriori for the fixed effect of time by sex interaction, cow age, and calf age at weaning and slaughter, and did not observe a significant effect; thus, those aforementioned factors were not included in the final statistical model as fixed effects. To evaluate the effect of maternal Se supplementation overall, we constructed a contrast that compared the average of the three maternal Se-supplementation groups versus the CTR group. In addition, we constructed three orthogonal contrasts, which compared each of the three maternal Se-supplementation groups versus the CTR group. For ordinal data, we used Fisher’s exact test (for independent samples) and McNemar’s test (for repeated samples). To detect at α = 0.05 with 80% power, a 0.5 carcass grade or carcass yield difference between treatment groups, we would have needed 16 calves per treatment group (assuming means were 2.0 and 2.5 and the standard deviation was 0.5).

Calypso v 8.84 [29] was used to analyze data from nasal swabs on the rarefied, total sum scaling (TSS) normalized ASV table. The diversity of bacterial communities within calves (alpha diversity) was determined using the Chao1 index, the observed ASVs, and the Shannon index. The observed ASVs and Chao1 estimate the number of different ASVs in a sample (richness). The Shannon index estimates the richness and the evenness in a sample by also including the number and relative abundance of different ASVs [30,31]. The diversity of bacterial communities between calves (beta diversity) was determined using the phylogeny-based UniFrac (weighted and unweighted) distance measures, which were then visualized using Principal Coordinate Analysis [32,33,34]. Unweighted UniFrac distance measures calculate whether treatment groups differ in the presence/absence of bacterial taxa. Weighted UniFrac distance measures calculate whether treatment groups differ in the presence and relative abundance of bacterial taxa.

QIIME2 was used to generate principal coordinate analysis (PCoA) plots for weighted and unweighted UniFrac distances. In order to find significant differences in microbial communities between groups, an ANOSIM (Analysis of Similarity) test was performed on the weighted and unweighted UniFrac distances. We compared bacterial taxa between groups using the non-parametric Mann–Whitney U test. The *p*- values were adjusted for multiple comparisons using Benjamini and Hochberg’s False Discovery Rate. We identified bacterial taxa that differed between CTR and TR calves using linear discriminant analysis effect size (LEfSe) with a cut-off of α = 0.05 and an LDA score >2.0 [35]. The statistical tests were all two-sided. Data are reported as least square mean ±SEM. Statistical significance is defined as a *p* ≤ 0.05 and a tendency at 0.05 < *p* ≤ 0.10.

## 3. Results

### 3.1. Effect of Supranutritional Se-Yeast Supplementation of Beef Cows on Whole-Blood Se Status of Their Calves at Weaning

**Figure 2:**
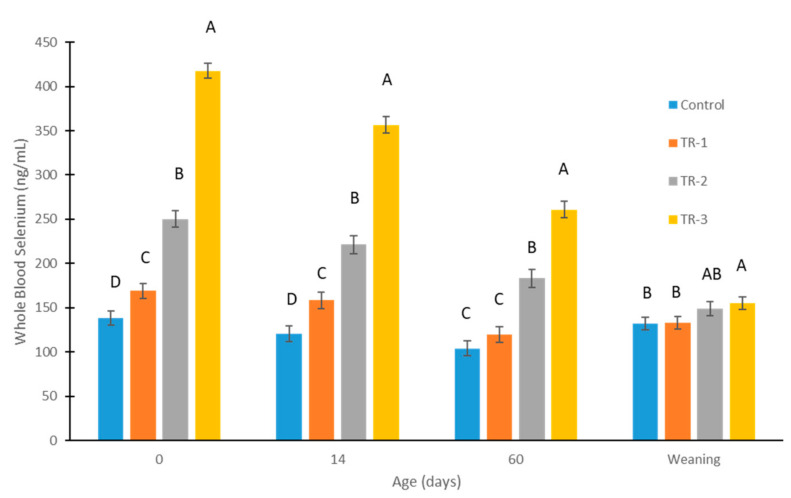
Whole-blood Se concentrations of calves measured at birth, 14 days, 60 days, and at weaning (6.5 to 8 months), from control cows and cows supplemented with Se-yeast boluses (105 mg Se/wk) during the first (TR-1), second (TR-2), and third (TR-3) trimester of gestation. Different letters above bars indicate which groups of calves differed significantly (*p* < 0.05) at that timepoint. The *p*-values for time, treatment groups, and their interaction were all <0.0001.

The concentrations of WB-Se at birth (0 days), 14 and 60 days of age, and at weaning are shown in Figure 2. The *p*-values for time, treatment groups, and their interaction were all <0.0001. Selenium supplementation of dams (supranutritional dosages) using weekly Se boluses during different trimesters was effective in increasing WB-Se concentration at birth in calves from all three Se-supplementation groups (all *p* < 0.01) compared with the calves of control dams (LSMean ± SEM; 138 ± 8 ng/mL). The effectiveness in increasing the WB-Se concentrations of calves was greatest when dams received Se supplementation in the last trimester (418 ± 8 ng/mL); medium increases were observed when dams received Se supplementation in the second trimester (250 ± 9 ng/mL); and the smallest increases were observed when dams received Se supplementation in the first trimester (169 ± 8 ng/mL).

The length of effectiveness in increasing the WB-Se concentrations of calves was greatest when dams received WB-Se concentrations in the last trimester. Higher WB-Se concentrations were observed until weaning in the calves of TR-3 dams (155 ± 7 ng/mL; *p* vs. control = 0.02). Smaller increases for a shorter time span were observed in the calves of TR-2 dams, as higher WB-Se concentrations were observed until 60 days of age (183 ± 10 ng/mL; *p* vs. control < 0.0001). The smallest increases for the shortest time span were observed in the calves of TR-1 dams, as higher WB-Se concentrations were observed until 14 days of age (158 ± 9 ng/mL; *p* vs. control = 0.003).

Female calves had higher WB-Se concentrations than male calves at birth (female: 259 ± 7 ng/mL vs. male: 229 ± 5 ng/mL; *p* < 0.0001). Sex differences were also observed at 14 days of age (female: 231 ± 8 ng/mL vs. male: 198 ± 6 ng/mL; *p* = 0.001), and at 60 days of age (female: 176 ± 8 ng/mL vs. male: 158 ± 6 ng/mL; *p* = 0.05). In contrast, sex differences were not significant at weaning (female: 146 ± 6 ng/mL vs. male: 139 ± 5; *p* = 0.37).

**Figure 3:**
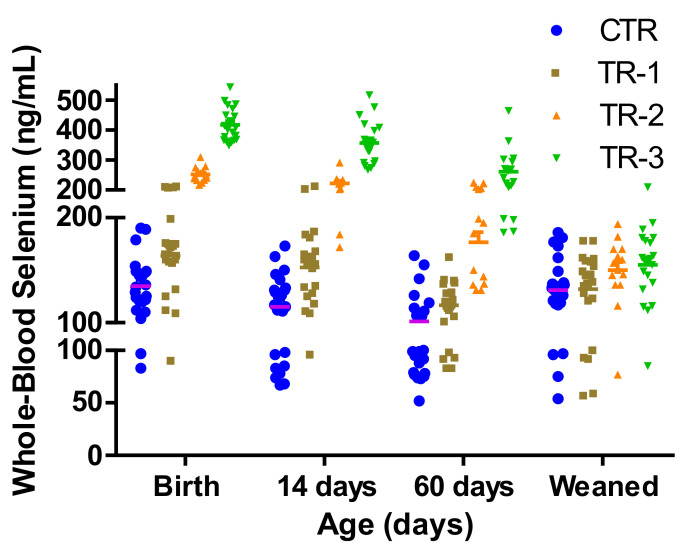
Whole-blood Se concentrations of individual calves measured at birth, 14 days, 60 days of age, and at weaning (6.5 to 8 months), from control cows and cows supplemented with Se-yeast boluses (105 mg Se/wk) during the first (TR-1), second (TR-2), and third (TR-3) trimester of gestation. Whole-blood Se concentrations < 50 ng/mL = clinical Se deficiency; WB-Se concentrations of 50–100 ng/mL below the reference range = subclinical Se deficiency; and WB-Se concentrations of 100–250 ng/mL (neonates) and 120–300 ng/mL (adults and growing calves) are in the reference range.

The concentrations of WB-Se decreased in CTR calves from birth to 60 days of age (138 ± 8 ng/mL to 104.2 ± 9 ng/mL; *p* = 0.0004) and then increased back to 132 ± 7 ng/mL at weaning. Many calves, especially male calves, without recent maternal Se supplementation had WB-Se concentrations indicating subclinical Se deficiency (Figure 3) [2,7].

A total of 11 of the 15 male CTR-calves, 6 of the 14 male TR-1 calves, 4 of the 8 female CTR calves, 3 of the 7 female TR-1 calves and 1 each of the 11 and 10 male TR-2 and TR-3 calves, respectively, during the repeated sampling times, had WB-Se concentrations below 100 ng/mL (Figure 3). As the time span between maternal Se supplementation and sampling increased, a larger number of TR calves became subclinically deficient (from two TR calves in the first 14 days to 11 TR calves during latter sampling times; McNemar test: *p* = 0.008).

### 3.2. Effect of Supranutritional Se-Yeast Supplementation of Beef Cows on Growth and Carcass Characteristics of Their Calves

The body weights at birth, 14 days, 60 days, weaning, and at slaughter of calves of the four calf groups were similar (group main effect: *p* = 0.21) and are shown in Table 1. The calves of TR-1 dams had lower birth weights than the calves from CTR dams (*p* = 0.04). The calves of TR-2 dams had lower weaning weights than the calves from CTR dams (*p* = 0.04), TR-1 dams (*p* = 0.06), and TR-3 dams (*p* = 0.02). Male calves at each sampling time had higher body or carcass weights than female calves (all *p* < 0.01) with the difference between weights increasing (sex-by-time interaction: *p* = 0.05). The calves of medium-frame-size cows had higher body weights than the calves of small- or medium-frame-size cows (*p* = 0.01), with no significant differences among sampling times (cow-frame-by-time interaction: *p* = 0.17).

The results of the carcass characteristics of the calves from the four groups are reported in Figure 1. The calves of TR-1 dams tended to have a higher proportion with select grade at slaughter (5 of 12 vs. 2 of 17; Fisher’s exact test: *p* = 0.09) and a lower proportion with a CAB certificate (0 of 13 vs. 5 of 13; Fisher’s exact test: *p* = 0.06) compared with the other two Se-supplemented groups (TR-2 and TR-3) combined. No other differences in carcass characteristics were observed (all *p* > 0.10).

### 3.3. Effect of Supranutritional Se-Yeast Supplementation of Beef Cows on Nasal Microbiome Genome of Their Calves at Weaning

**Figure 4:**
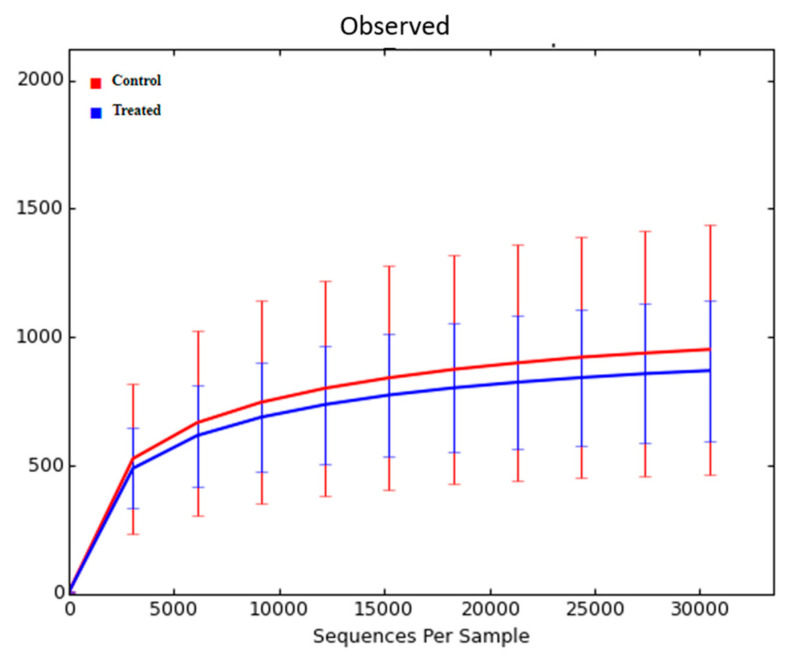
Rarefaction analysis of 16S rRNA amplicon sequence variants (observed ASV’s) obtained from nasal swabs of calves at weaning. Data from 11 calves from control cows and cows supplemented with Se-yeast boluses (105 mg Se/wk), during the first (TR-1) trimester of gestation, that had WB-Se in the range of 0.06 to 0.13 ng/mL were compared with 10 calves from cows supplemented with Se yeast during the second (TR-2) and third (TR-3) trimesters of gestation that had WB-Se in the range of 0.15 to 0.21 ng/mL.

The nasal microbiome was collected at weaning. Sequencing of the bacterial 16S rRNA gene in the samples yielded 3,062,213 quality sequences (76,555 ± 22,297, mean ± SD). Rarefaction analysis was performed at a depth of 30,450 sequences. There was no significant difference in nasal microbiome diversity in calves at weaning based on the treatment of dams with Se-yeast boluses (total dosage: 105 mg Se/wk) during the first (TR-1), second (TR-2), and third (TR-3) trimester of gestation combined, compared with the control calves (data not shown). There was also no difference in nasal microbiome diversity among calves of the Se-treatment groups (TR-1 vs. TR-2 vs. TR-3) at weaning (data not shown). Subsequently, we reanalyzed the data from 11 calves from control cows and cows supplemented, during the first (TR-1) trimester of gestation, that had WB-Se in the range of 0.06 to 0.13 ng/mL, and compared the with 10 calves from TR-2 and TR-3 groups that had WB-Se in the range of 0.15 to 0.21 ng/mL. There was no significant difference in nasal microbiome alpha diversity in calves at weaning based on their WB-Se concentration in these two ranges (Figure 4). This was true for all three measures of alpha diversity (observed ASV’s, Chao1, and Shannon index) (Table 2).

**Figure 5:**
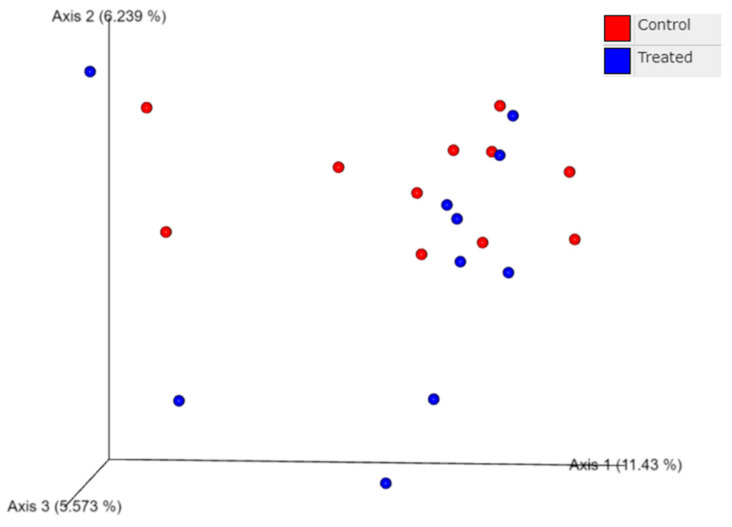
Principal coordinate analysis of unweighted UniFrac distances obtained from nasal swabs of calves at weaning. Data from 11 calves from CTR cows and cows supplemented with Se-yeast boluses (105 mg Se/wk), during the first (TR-1) trimester of gestation, that had WB-Se in the range of 0.06 to 0.13 ng/mL were compared with 10 calves from cows supplemented with Se yeast during the second (TR-2) and third (TR-3) trimesters of gestation that had WB-Se in the range of 0.15 to 0.21 ng/mL.

The nasal microbiome from the nasal swabs of calves at weaning also did not differ for beta diversity measures, as shown by principal coordinate analysis of UniFrac distances. Beta diversity looks at differences in the bacterial species between two groups of calves. Unweighted UniFrac distance measures differences in the number of ASV’s present, whereas the weighted UnifFrac distance accounts for differences in the number of bacterial species as well as the relative abundance of different bacterial species [32,34]. Principal coordinate analysis calculates the distances between all the samples and then displays the distances in a 2D or 3D space [33]. Principal coordinate analysis focuses on variation within treatment groups, similarly to standard deviation. There was no distinct clustering of calves at weaning based on the treatment of dams with Se-yeast boluses (105 mg Se/wk) during the first (TR-1), second (TR-2), and third (TR-3) trimester of gestation combined compared with the control calves (data not shown). There was also no distinct clustering of calves among calves of the Se-treatment groups (TR-1 vs. TR-2 vs. TR-3) at weaning (data not shown). The clustering of data from 11 calves from the control cows and cows supplemented with Se-yeast boluses (105 mg Se/wk), during the first (TR-1) trimester of gestation, that had WB-Se in the range of 0.06 to 0.13 ng/mL was compared with 10 calves from cows supplemented with Se yeast during the second (TR-2) and third (TR-3) trimesters of gestation that had WB-Se in the range of 0.15 to 0.21 ng/mL (Figure 5). The ANOSIM values (R_unweighted_ = 0.0258, *p* = 0.22; R_weighted_ = 0.0078, *p* = 0.38) of UniFrac distances were not significant.

### 3.4. Effect of Supranutritional Se-Yeast Supplementation of Beef Cows on Nasal Microbiota of Their Calves at Weaning

**Figure 6:**
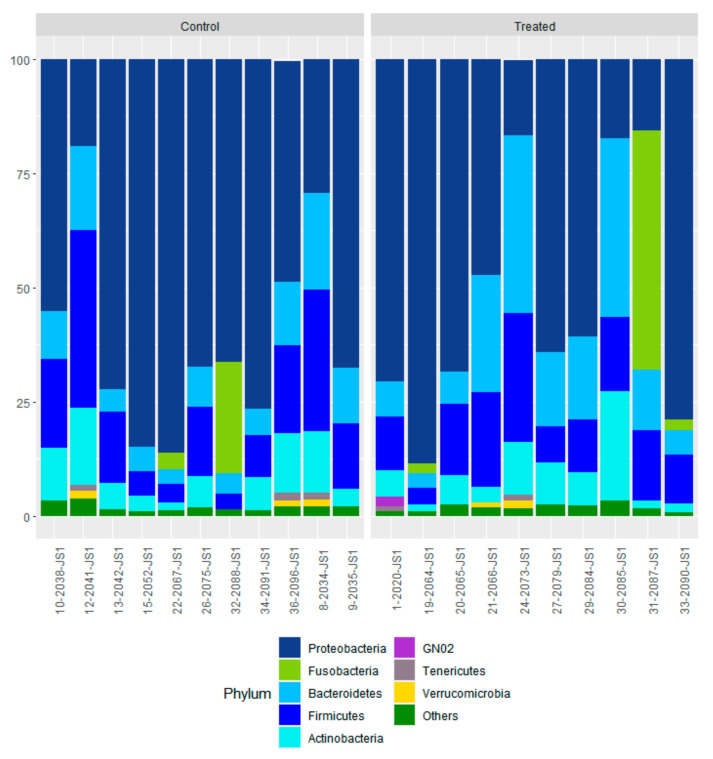
Nasal microbiota profiles of control calves at weaning. Data from 11 calves from control cows and cows supplemented with Se-yeast boluses (105 mg Se/wk), during the first (TR-1) trimester of gestation, that had WB-Se in the range of 0.06 to 0.13 ng/mL were compared with 10 calves from cows supplemented with Se yeast during the second (TR-2) and third (TR-3) trimesters of gestation that had WB-Se in the range of 0.15 to 0.21 ng/mL.

**Figure 7:**
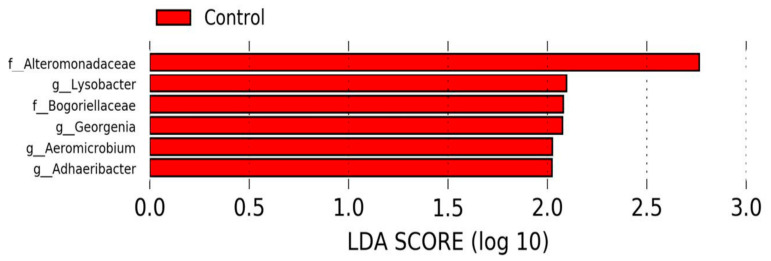
Differentially abundant taxa identified using LEfSe showing taxa that were enriched in calves from control dams at weaning (α = 0.05, LDA score > 2.0).

We detected 32 bacterial phyla in nasal swabs at the time of weaning. The profiles of the eight most abundant bacterial phyla for individual calves are shown in Figure 6. The major nasopharynx phyla (with relative abundance > 1%) were Proteobacteria (67.1% CTR and TR-1; 62.4% TR-2 and TR-3), Firmicutes (15.3% CTR and TR-1; 13.5% TR-2 and TR-3), Bacteroidetes (8.7% CTR and TR-1; 14.8% TR-2 and TR-3), and Actinobacteria (6.8% CTR and TR-1; 6.1% TR-2 and TR-3). Based on LEfSe, the bacterial families (Alteromonadaceae and Bogoriellaceae) and the genera (Adhaeribacter, Aeromicrobium, Georgenia, and Lysobacter) were enriched in the control group (Figure 7). However, when individual bacterial taxa were compared between the two groups using a Mann–Whitney U test and adjusted for multiple comparisons, there were no significant differences in any bacterial group (*p* > 0.05) (Table S1).

## 4. Discussion

The objective of this study was to evaluate the effect of maternal supranutritional Se supplementation during various trimesters of pregnancy on the Se status, growth, nasal microbiome at weaning, and carcass characteristics of their calves. To our knowledge, this is the first study which examines the impact of maternal supranutritional Se supplementation during various trimesters of pregnancy on calf performance and health. In two previous studies, we supplemented dairy [16] and beef [36] cattle, respectively, for the last 8 or 8 to 12 weeks of pregnancy, respectively, with either a single weekly Se-yeast top dressing of their total mixed ration (105 mg Se yeast/wk) or with Se-biofortified alfalfa hay containing 5.17 mg Se/kg of DM (equivalent to 57.5 mg Se/cow daily), respectively. Maternal, supranutritional Se supplementation of dairy or beef cows during the last trimester of pregnancy increased WB-Se concentrations in their calves by 150% and 300%, respectively. In the current study, cows were treated once weekly with Se via a selenized yeast bolus (105 mg Se yeast/wk) and dosing was consistently maintained for 13 weeks. Because we achieved similar WB-Se concentrations in calves at birth in the current study, we conclude that it is possible to substitute once-weekly treatment of dams with Se-yeast boluses for daily feeding of Se-enriched alfalfa hay and achieve similar WB-Se concentrations of calves at birth.

In our previous studies, we did not follow WB-Se concentrations of calves beyond the first 48 h in beef calves [36] or 14 days in dairy calves [16]. In the current study, we report that higher WB-Se concentrations in calves of TR-3 dams can persist until weaning. Thus, we conclude it is possible to provide calves with Se-body reserves through maternal, supranutritional Se supplementation. In the current study, Se supplementation of dams during the last trimester of pregnancy was better suited than during earlier trimesters of pregnancy to accumulate Se-body reserves in calves. In support, we observed higher muscle Se concentrations in calves of TR-3 cows compared with calves from CTR or TR-1 and TR-2 cows [37].

The current guidelines for WB-Se concentrations in calves (0 to 30 days of age) suggest that WB-Se concentrations of 100–250 ng/mL are adequate [2]. We observed several calves, especially male calves, that had subclinically deficient WB-Se concentrations during the nursing period. We conclude that postpartum Se supplementation of calves is very important by 60 days, as Se attained during pregnancy is waning and intake of supplemental Se provided by Se-fortified salt (120 mg/kg Se; provided ad libitum) is insufficient to maintain WB-Se concentrations in the desired range. Given the faster growth rates of male compared with female calves, male calves may have higher Se requirements.

Maternal, supranutritional Se-yeast supplementation of TR-3 cows did not impact growth performance. We previously reported higher birth weights of dairy calves whose dams were supplemented weekly with Se yeast for the last 8 weeks of pregnancy [16], but not in the beef calves whose dams received biofortified alfalfa hay supplementation [36]. Interestingly, the calves from TR-1 dams in the current study had lower birth weights compared with the calves from CTR dams. This was likely because the genes involved in muscle development (specifically muscle-structure-related genes) were downregulated in the TR-1 calves compared with the CTR calves [37]. In addition, the TR-1 calves tended to have a higher proportion graded select at slaughter compared with the other two Se-supplemented groups combined. We conclude that maternal Se supplementation during the first trimester may not benefit the growth performance of calves.

Given the lower weaning weights of calves from TR-2 cows, maternal Se supplementation during the second trimester also may not be beneficial for the muscle growth of calves. The hot carcass weight of the calves from TR-2 cows tended to be lower compared with the calves from CTR cows (*p* = 0.07). In addition, we previously reported that the genes involved in collagen formation were downregulated in the muscle of newborn beef calves of TR-2 vs. CTR cows [37]. Given that the growth characteristics of the calves from TR-1 and TR-2 cows were inferior to the calves of CTR cows, supranutritional maternal Se supplementation throughout pregnancy is not recommended. Thus, we recommend maternal, supranutritional Se-yeast supplementation during the last trimester as the best choice. This recommendation is supported by the fact that the calves of TR-3 vs. CTR calves had upregulated myosin- and actin-filament-associated genes in their *longissimus dorsi* muscle, indicating a potential benefit for optimal muscle function and contraction [37].

We have previously reported, in three clinical trials [9,11,12], that feeding Se-biofortified alfalfa hay to recently weaned calves during the preconditioning period improved not only growth but also health in the feedlot and carcass characteristics. We did not observe similar improvements in performance with maternal, supranutritional Se supplementation during the various pregnancy trimesters in the current study. When comparing WB-Se concentrations among the studies, three potential reasons for differences in performance were observed: (1) Large differences in WB-Se concentrations among the studies. In the three former studies, the WB-Se concentrations of Se-supplemented weaned beef calves were between double and triple those of non-supplemented calves. In contrast, group differences were +17% at weaning between TR-3 vs. CTR calves in the present study; (2) Low WB-Se concentrations in the CTR-calves. Improvements in body weight, health, or carcass characteristics were observed in the two studies that had unsupplemented calves with a WB-Se concentration < 100 ng/mL [9,11]; (3) BRD challenges in the feedlot. Improvements in body weight, health, and carcass characteristics were observed in the study, with calves having the lowest WB-Se concentrations and the most severe BRD outbreak. The BRD outbreak killed 45% of the CTR calves and none of the calves that received Se-biofortified hay at 18.8 mg Se/calf per day [10]. In contrast, only two calves did not reach slaughter weight in the current study (one male each from the CTR and TR-1 groups). We conclude that additional Se supplementation of calves might be needed to optimize health and growth performance in the feedlot, specifically in the event of BRD outbreaks. Others have also shown that steers from cows supplemented with organic Se tended to enter the feedlot heavier compared with steers from cows supplemented with inorganic Se [38]. Maternal Se sources had no effect on hot carcass weight, yield grade or quality grade in that study [38]. Pre-weaning performance also was not altered [39]. In another study, beef cattle that received nutraceutical support with organic Se, live yeast, and mannan oligosaccharides during the first 30 days after entering the feedlot tended to have decreased bovine respiratory disease occurrence, increased final body weight, and increased average daily gain at day 30, but there was no difference in terms of carcass characteristics [40]. That study also supported the use of Se in beef cattle early in the feedlot cycle to improve the ability of cattle to react against pathogens, as well as increase feed efficiency and growth performances during the feedlot period [40].

Diversity of the nasal microbiota can play an important role in preventing overgrowth of BRD-causing pathogens such as mycoplasma. In our previous study, 14 of 30 calves developed, upon entry to the feedlot, an overgrowth of mycoplasma (15–60% of total nasal bacteria); this number was lower in calves fed Se-biofortified alfalfa hay during the preconditioning period [11]. In our previous studies, feeding Se-biofortified alfalfa hay during the preconditioning period was associated with an increase in nasal microbiota diversity in a Se-supplementation dose-dependent manner. Thus, we concluded that increasing nasal microbiota diversity by feeding Se-biofortified hay during the preconditioning period may improve health by preventing overgrowth of BRD-causing pathogens such as mycoplasma. In the current study, we did not observe differences in the diversity of nasal microbiota. Potential reasons for the different results could be: (1) Differences in WB-Se concentrations. In the current study, WB-Se concentrations were only 17% higher in TR-3 vs. CTR calves compared to 3-fold differences in previous studies; (2) differences in microbial diversity in CTR calves. The calves of CTR cows had, in the current study, higher measures of microbial diversity at weaning than in the previous study [11]. We conclude that maternal, supranutritional Se supplementation is insufficient to increase nasal microbial diversity. Additional Se supplementation of calves is needed during the backgrounding period to observe differences in nasal microbiota.

Limitations of this study include the fact that that not all calves could be followed through the feedlot to assess performance, as some female calves were retained as seed stock, and male calves were also retained for a production class (Figure 1). The favorable retention of TR-3 animals may have prevented us from observing more favorable results for carcass data. Additionally, we did not achieve WB-Se concentrations at weaning as high as those seen in previous studies, when we supplemented dams with Se-enriched forage during pregnancy rather than Se-yeast boluses. We do not know at what WB-Se concentration ranges beneficial changes in the nasal microbiome at weaning, and subsequent meat characteristics at slaughter, are observed.

## 5. Conclusions

In conclusion, we cannot rely on Se supplementation of the cow during pregnancy to carry the calf through to the feedlot. Ultimately it is best to provide Se supplement to calves again at weaning, as we did in our previous studies, in order to see a benefit in nasal microbiome abundance and diversity. The timing of Se supplementation appears critical, as WB-Se concentrations and the physiological effects of Se attenuate the longer the time between the last Se application and assessment. Thus, calves should be fed Se-biofortified forages again at weaning in order to diversify the nasal microbiome prior to entering the feedlot.

## Figures and Tables

**Table 1:** Postpartum body weights and hot carcass weights (kg; LSmean) for calves from control dams and from dams supplemented with Se-yeast boluses (105 mg Se/wk) during the first (TR-1), second (TR-2), and third (TR-3) trimester of gestation.

Body Weight	Control	TR-1	TR-2	TR-3	SEM	*p* Value
(kg)	(*n* = 23)	(*n* = 21)	(*n* = 14)	(*n* = 20)		
0 days (birth)	39.7 ^a^	36.8 ^b^	40.0 ^a^	38.7 ^ab^	1.2	0.22
14 days	56.1	53.1	52.5	55.5	2.0	0.31
60 days	110.2	108.6	102.9	110.8	4.0	0.38
224 days (weaning)	295.7 ^a^	294.4 ^ab^	272.1 ^b^	299.9 ^a^	9.6	0.10
474 days (carcass)	411.6	404.7	383.2	391.5	13.1	0.26

^a,b^ Means with different superscripts within a row indicate significant pairwise differences (*p* < 0.05). SEM: standard error of mean (the largest of the 4 SEMs are shown). *p*-values indicate overall group differences.

**Table 2:** Measures of alpha diversity (mean ± SD) in nasal microbiome genome from nasal swabs of calves at weaning. Data from 11 calves from control cows and cows supplemented with Se-yeast boluses (105 mg Se/wk), during the first (TR-1) trimester of gestation, that had WB-Se in the range of 0.06 to 0.13 ng/mL were compared with 10 calves from cows supplemented with Se yeast during the second (TR-2) and third (TR-3) trimesters of gestation that had WB-Se in the range of 0.15 to 0.21 ng/mL.

	Calves from Control and TR-1 Dams (*n* = 11)	Calves from TR-2 and TR-3 Dams (*n* = 10)	*p*-Value
Chao1	1028 ± 564.4	931.1 ± 324.4	0.63
Observed ASV	952.5 ± 510.0	870.1 ± 290.6	0.65
Shannon Index	7.4 ± 1.6	7.2 ± 0.9	0.76

## Data Availability

All relevant data are within the paper and its Supplementary Information files.

## Supplementary Materials

The following supporting information can be downloaded at: https://www.mdpi.com/article/10.3390/ani12111360/s1, Table S1: Relative percentages of the most abundant bacterial groups annotated to the various phylogenetic levels (phylum, class, order, family, genus). Median and range of the relative abundance of bacteria are shown for 11 calves from control cows and cows supplemented with Se-yeast boluses (105 mg Se/wk), during the first (TR-1) trimester of gestation, that had WB-Se in the range of 0.06 to 0.13 ng/mL, compared with 10 calves from cows supplemented with Se-yeast during the second (TR-2) and third (TR-3) trimesters of gestation that had WB-Se in the range of 0.15 to 0.21 ng/mL.
